# Dynamic changes in glymphatic function in reversible cerebral vasoconstriction syndrome

**DOI:** 10.1186/s10194-024-01726-1

**Published:** 2024-02-05

**Authors:** Chia-Hung Wu, Yu Kuo, Yu-Hsiang Ling, Yen-Feng Wang, Jong-Ling Fuh, Jiing-Feng Lirng, Hsiu-Mei Wu, Shuu-Jiun Wang, Shih-Pin Chen

**Affiliations:** 1 Department of Radiology, Taipei Veterans General Hospital, No.201, Sec. 2, Shipai Rd., Taipei, Taiwan; 2 School of Medicine, National Yang Ming Chiao Tung University, No. 155, Sec. 2, Linong St, Taipei, Taiwan; 3 Department of Nuclear Medicine, Taipei Veterans General Hospital, No.201, Sec. 2, Shipai Rd., Taipei, Taiwan; 4 Department of Neurology, Neurological Institute, Taipei Veterans General Hospital, No.201, Sec. 2, Shipai Rd., Taipei, Taiwan; 5 Brain Research Center, National Yang Ming Chiao Tung University, No. 155, Sec. 2, Linong St., Taipei, Taiwan; 6 Institute of Clinical Medicine, National Yang Ming Chiao Tung University, No. 155, Sec. 2, Linong St., Taipei, Taiwan; 7 Division of Translational Research, Department of Medical Research, Taipei Veterans General Hospital, No.201, Sec. 2, Shipai Rd., Taipei, Taiwan

**Keywords:** Reversible cerebral vasoconstriction syndrome (RCVS), Healthy controls (HCs), Glymphatics, Transcranial color-coded duplex sonography (TCCS), Diffusion-tensor imaging along the perivascular space (DTI-ALPS) index, The six-item Headache Impact Test (HIT-6)

## Abstract

**Background:**

The pathophysiology of the reversible cerebral vasoconstriction syndrome (RCVS) remains enigmatic and the role of glymphatics in RCVS pathophysiology has not been evaluated. We aimed to investigate RCVS glymphatic dynamics and its clinical correlates.

**Methods:**

We prospectively evaluated the glymphatic function in RCVS patients, with RCVS subjects and healthy controls (HCs) recruited between August 2020 and November 2023, by calculating diffusion-tensor imaging along the perivascular space (DTI-ALPS) index under a 3-T MRI. Clinical and vascular (transcranial color-coded duplex sonography) investigations were conducted in RCVS subjects. RCVS participants were separated into acute (≤ 30 days) and remission (≥ 90 days) groups by disease onset to MRI interval. The time-trend, acute stage and longitudinal analyses of the DTI-ALPS index were conducted. Correlations between DTI-ALPS index and vascular and clinical parameters were performed. Bonferroni correction was applied to vascular investigations (*q* = 0.05/11).

**Results:**

A total of 138 RCVS patients (mean age, 46.8 years ± 11.8; 128 women) and 42 HCs (mean age, 46.0 years ± 4.5; 35 women) were evaluated. Acute RCVS demonstrated lower DTI-ALPS index than HCs (*p* < 0.001) and remission RCVS (*p* < 0.001). A continuously increasing DTI-ALPS trend after disease onset was demonstrated. The DTI-ALPS was lower when the internal carotid arteries resistance index and six-item Headache Impact test scores were higher. In contrast, during 50–100 days after disease onset, the DTI-ALPS index was higher when the middle cerebral artery flow velocity was higher.

**Conclusions:**

Glymphatic function in patients with RCVS exhibited a unique dynamic evolution that was temporally coupled to different vascular indices and headache-related disabilities along the disease course. These findings may provide novel insights into the complex interactions between glymphatic transport, vasomotor control and pain modulation.

**Supplementary Information:**

The online version contains supplementary material available at 10.1186/s10194-024-01726-1.

## Background

Reversible cerebral vasoconstriction syndrome (RCVS) is a neurovascular disorder with complicated pathophysiologies [1, 2]. RCVS characteristics include recurrent thunderclap headaches and vasoconstriction reversibility, [3, 4] and may complicate with cerebral hemorrhages, subarachnoid hemorrhages, infarctions and posterior reversible encephalopathy syndrome [3, 4]. Autonomic dysfunction, [5] increased oxidative stress [6] and disrupted blood–brain barrier integrity [7–9] have been proposed to contribute to the disease.

The glymphatic-meningeal lymphatic system plays a significant role in cerebrospinal fluid solute clearance [8]. Its impairments have been linked to Parkinsonism, [10] strokes, [11] and Alzheimer’s disease [12] and several other neurological disorders [13–15]. However, investigations focusing on RCVS glymphatics remain scarce.

The diffusion tensor imaging along the perivascular space (DTI-ALPS) index, adopting DTI data to calculate the naturally perpendicular directions of the perivascular space diffusivity at axial ventricular level, quantifies glymphatics noninvasively [16]. This noninvasive glymphatic quantification, without intrathecal [17] or intravenous [8, 10, 18] tracer administration, has been shown to be correlated with multiple attributes in several physiological conditions and diseases, including headache, [19] sleep, [20] Alzheimer’s disease, [12] and neuromyelitis optica, [21] suggesting its potential utility in investigating the glymphatic function in neurological disorders. In the glymphatic hypothesis, the interstitial fluid assists the intracranial solutes excretion and travels along the perivascular interstitial spaces [22, 23]. Since the DTI-ALPS index calculates the perivascular space fluid diffusivity [16], its value reflects the interstitial flow ability to excrete the solutes [22]. Therefore, we took the advantage of this particular index to investigate the interstitial flow functions that contribute to the intracranial solute removal.

The primary outcome of this study was to investigate the glymphatic changes in subjects with RCVS in different disease stages using the DTI-ALPS index. The secondary aims were to evaluate the potential correlations of the DTI-ALPS index with vascular parameters on transcranial color-coded duplex sonography (TCCS) and clinical parameters.

## Methods

### Ethics

All study subjects provided written informed consent before enrollment, and the study protocols were approved by the Institutional Review Board of Taipei Veterans General Hospital (2019–02-013A & 2021–02-018C). All investigations were conducted according to the principles of the Declaration of Helsinki.

### Study subjects

In this study, HCs and subjects with RCVS were prospectively enrolled between August 2020 and November 2023. The HCs were recruited from the nearby communities. Any subjects with known cancer history, psychiatric disorders, major neurological diseases, personal or family history (within third degree relatives) of moderate to severe migraine-like headache were to be excluded. The major neurological diseases included ischemic stroke, [11] hemorrhagic stroke, [24] demyelination, or neuroinflammatory diseases, [10, 15, 25] intracranial tumors, [26] and neurodegenerative diseases [10, 27] since they had been reported to have glymphatic impairments. The psychiatric disorders included sleep disorders [28] and mood disorders, [29, 30] which were also linked to glymphatic dysfunctions. As some migraine subjects, particularly those with chronic migraine, may have glymphatic dysfunction, [19, 31, 32], subjects with potential or known migraine were not qualified to be HCs in this study. Since migraine holds strong family tendency, [33] subjects with known family history of moderate to severe migraine or migraine-like headache were excluded from the HCs (Supplementary Table 1 in Additional file 1). The RCVS subjects were recruited from the Headache Clinics and Emergency Department at a single state-run tertiary referral center. The RCVS diagnosis was based on the criteria used in the previous studies [8, 9, 34]. These criteria were consistent with those in the International Classification of Headache Disorders, third version (ICHD-3) [35]. All RCVS subjects completed a thorough headache questionnaire, which included the six-item Headache Impact Test (HIT-6) [36] to evaluate headache-related disabilities (Supplementary Table 2 in Additional file 1). The enrolled RCVS subjects failed to demonstrate the reversibility of MRA vasoconstrictions or under any medications other than nimodipine were excluded.

All the RCVS subjects were interviewed by the headache specialists (SPC, SJW and YFW). We inquired all the RCVS patients with detailed headache information, including but not limited to headache characteristics, onset time/date, duration, accompanying symptoms, the use of painkillers, the effects of painkillers and previous headache history. In our practice, all RCVS subjects were treated with nimodipine upon the diagnosis [37]. The dosage was meticulously adjusted by close blood pressure monitoring to avoid systolic blood pressure < 100 mmHg. [38] Subjects under medical treatments other than nimodipine were not enrolled and we did not administrate medications other than nimodipine during the disease course. Given the varied timepoints of patient enrollment relative to disease onset, the diffusion tensor imaging (DTI) for most patients was conducted concurrently with the diagnostic MRI, while for others it was performed after diagnostic ascertainment. Consequently, not every DTI during the acute phase was executed prior to nimodipine administration. Though this variation in enrollment timing introduces analytical challenges, we leveraged it as a unique opportunity to explore the dynamics of glymphatic function and to investigate the potential impact of nimodipine during the acute phase.

**Fig. 1:**
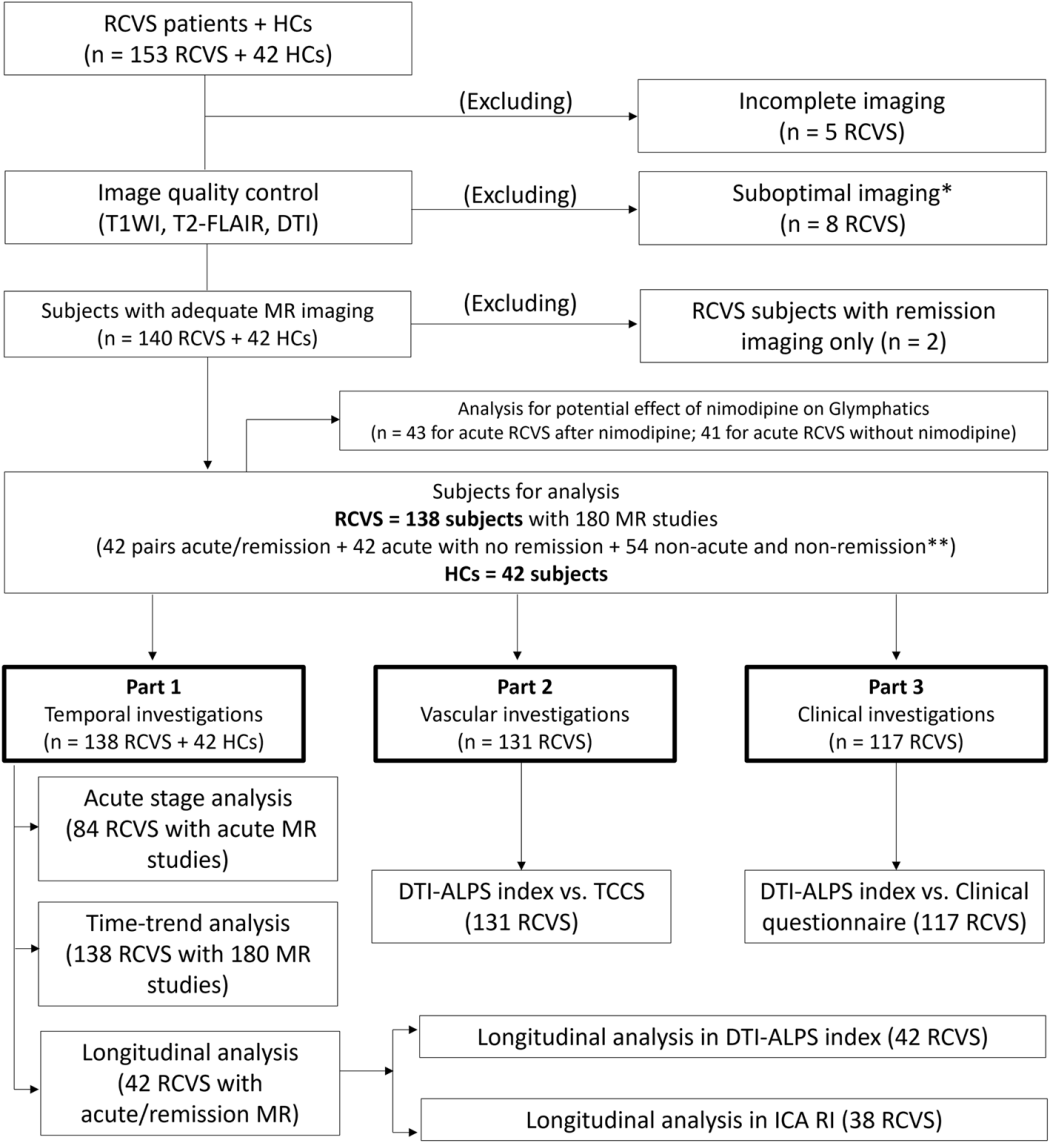
Flow diagram of the subject enrollments. *Suboptimal imaging: 4 with metallic artifacts due to dental prothesis; 3 with asymmetric scans; 1 with file storage error. **Onset to MRI interval: acute ≤ 30 days; remission ≥ 90 days; non-acute and non-remission are those > 30 and < 90 days. DTI = Diffusion tensor imaging; DTI-ALPS = Diffusion-tensor imaging along the perivascular space; HC = Healthy control; HIT-6 = The six-item Headache Impact Test; ICA = Internal carotid artery; MR = Magnetic resonance; RCVS = Reversible cerebral vasoconstriction syndrome; RI = Resistance index; TCCS = Transcranial color-coded duplex sonography; T1WI = T1-weighted imaging; T2-FLAIR = T2-fluid-attenuated inversion recovery

This study included three study parts. Part 1 was composed of acute stage, time-trend and longitudinal analyses. The enrolled subjects were separated into two groups based on their disease onset to MRI interval (acute ≤ 30 days; remission ≥ 90 days) [9]. Those who agreed to be enrolled in the longitudinal analysis and were approached in their acute stages were included in the longitudinal analysis. The first MR session for the subjects in the longitudinal analysis was conducted ≤ 30 days and the follow-up MR session would be arranged ≥ 90 days. Parts 2 and 3 included clinical and vascular investigations with headache questionnaires and TCCS data, respectively (Fig. 1).

### Imaging protocols

All MR exams were conducted with the same 3-T (T) MRI machine (MR750, GE Healthcare, Chicago, Il). DTI was performed with a b-value of 1000 s/mm^2^ (repetition time/echo time = 7500.0/58.10 ms; diffusion gradient encoding in 30 directions) [16]. Three-dimensional (3D) time-of-flight MR angiography (TOF-MRA; repetition time/echo time = 25.00/2.90 ms, 1 mm section thickness; multislab acquisitions) and susceptibility-weighted imaging (Susceptibility Weighted Angiography; SWAN; repetition time/echo time = 41.00/23.64 ms, 2 mm section thickness) were performed to evaluate both large and small vessels.

High-resolution and 3D imaging to exclude potential structural lesions or vascular complications, including T2-fluid-attenuated inversion recovery (T2-FLAIR; repetition time/echo time/inversion time = 6,000/128/1870 ms; 1 mm slice thickness) and T1WI (3D-T1-ultrasfast gradient echo: repetition time/echo time/inversion time = 9.18/3.68/450 ms, 1 mm section thickness), were performed in the same session.

### Imaging analysis

The DTI, T1WI, T2-FLAIR, TOF-MRA and SWAN data were acquired, and detailed inspections of the images were performed with the picture archiving and communication system (PACS; SmartIris, version 2.1.0.11, The Taiwan Electronic Data Processing Co.). Any suboptimal images, including incomplete scans and those with potential artifacts, intracranial tumors or other potential factors that may interfere with further analyses, were excluded (Fig. 1).

The data were then transferred to the VolumeViewer (version 11.0, GE Healthcare, Chicago, Il) platform. On this platform, automatic synchronization and motion corrections were performed and imaging quality controls were conducted again. The calculations of the diffusivities in the x-, y- and z-directions were performed with the DTI-Advanced toolbox in VolumeViewer. Fixed spherical regions of interest (ROIs) with 2.5 mm diameters were placed onto the areas of the projection and association fibers at the ventricular levels. The differentiation of the areas was based on the color-mapped anisotropy generated automatically by the software. We adopted T2-FLAIR, TOF-MRA and SWAN imaging as references, which were automatically synchronized with the DTI maps, to avoid ROI placements onto the regions with WMHs on T2-FLAIR or overt vascular structures on TOF-MRA or SWAN. These ROIs were marked by the two neuroradiologists. In order to place the ROIs onto the accurate and consistent anatomical locations, we chose to mark at the upper ventricular levels (Supplementary Figure 1 in Additional file 1) on axial section in all the subjects. The two interpreters marked the ROIs separately and were blind to the clinical information of the given subjects. The final values were adopted based on the consensus if discrepancy existed. We used the acquired values to calculate the DTI-ALPS index as the quotient of the mean of the x-projection and x-association areas over the mean of the y-projection and z-association areas [16].

The TCCS vascular parameters of the middle cerebral arteries (MCAs) and distal extracranial internal carotid arteries (ICAs) were recorded and calculated in RCVS subjects. These parameters included mean flow velocities, pulsatility index (PI), and resistance index (RI). The MCA evaluations included proximal and distal M1 segments separated by a 20-mm distance [3, 9]. Since the PIs in large vessels decrease from proximal to distal segments [39] and are affected by the locations of vasoconstrictions, the mean PI was also calculated in the distal and proximal segments of both MCAs [3, 9]. The mean RI (Supplementary Table 3 in Additional file 1) was calculated with similar methods. The mean RI ratio in the distal and proximal M1 was calculated as the ratio of the distal RI to proximal RI [3, 9]. The Lindegaard index (LI) was also calculated as the ratio of the mean flow velocity of the MCA to that of the ipsilateral ICA. The averaged values of bilateral TCCS vascular parameters were adopted in the subsequent analyses.

### Statistical analysis

Descriptive statistics were reported as percentages and means ± standard deviations (SDs) or medians with interquartile ranges (IQRs) when appropriate. The sex and age differences between groups were evaluated by Chi-squared tests and t tests respectively. Normality was evaluated with the Kolmogorov–Smirnov test, revealing that the DTI-ALPS index was not normally distributed (*p* < 0.001). This study included three study parts. In the acute stage analysis in Part 1 comparing the DTI-ALPS differences between the acute RCVS and HCs were evaluated by Mann‒Whitney U tests. In the longitudinal analysis comparing the DTI-ALPS index and vascular resistance (RI) were evaluated by related-samples Wilcoxon signed rank tests. In Parts 2 and 3, the correlations between the DTI-ALPS index and clinical and TCCS vascular parameters were explored by Spearman's rank correlation coefficients. To investigate the potential effects of the temporal changes of glymphatic function, the analysis in Part 2 was also conducted in RCVS subjects grouped by the given time intervals depicted in Part 1. Bonferroni correction was applied to the Part 2 since we evaluated the potential correlations between DTI-ALPS index with each of the 11 vascular parameters (*q* value = 0.05/11). In the Part 3, any significant results were further evaluated again as covariant in RCVS subjects and the differences between the selected subjects regarding the significant clinical parameters were tested by quade nonparametric analysis of covariance with age adjustments when needed. The interrater agreements of the DTI-ALPS index were evaluated by single measurements of the intraclass correlation coefficients. The above analyses were performed using Statistical Product and Service Solutions (SPSS, IBM Corp.) statistical software package, version 28.0.

## Results

### Study subjects

A total of 195 subjects (153 RCVS and 42 HCs) were enrolled in this study (Fig. 1). After exclusion of inadequate imaging (*n* = 13 RCVS) and RCVS subjects with only remission imaging (*n* = 2), 138 RCVS subjects with 180 MR studies (including 42 subjects with 42 pairs of acute and remission MR, 42 subjects with acute MR only, and 54 subjects with MR performed in-between acute and remission stages) and 42 HCs data were analyzed in this study (Table 1). In Part 1, all the 138 RCVS and 42 HCs data were analyzed for time-trend investigations. In addition, the data from all the 84 RCVS subjects with acute stage MRI and 42 HCs were compared. Then, data from 42 RCVS subjects with both acute and remission MR studies were analyzed for the longitudinal analysis. Among all the 138 RCVS subjects, those with available TCCS data (*n* = 131) were analyzed in Part 2 and those with compete questionnaire (*n* = 117) were analyzed in Part 3. The intraclass correlation coefficient of the DTI-ALPS index of all subjects was 0.829.

**Table 1:** Demographics of all subjects

	RCVS subjects	Healthy controls
Total number of subjects	138	42
Total number of MR scans	180	42
Clinical characteristics		
Age (y)	46.8 ± 11.8	46.0 ± 4.5
Number of females	128 (92.7%)	35 (83.3%)
Menopause^a^	81 (69.2%)	23 (65.7%)
Symptoms at disease onset		
Thunderclap headache	138 (100%)	-
Seizure	0	-
Headache characteristics		
Nausea/vomiting	26 (18.8%)	-
Unilaterality	107 (77.5%)	-
Pulsating	60 (43.5%)	-
Photophobia	22 (15.9%)	-
Phonophobia	43 (31.2%)	-
Timing intervals		
MR to headache questionnaire interval (days, *n* = 117)	0	-
MRI to TCCS interval (days, *n* = 131)	3.4 ± 2.1	-

*MR* Magnetic resonance, *MRI* Magnetic resonance imaging, *n* Number(s), *RCVS* Reversible cerebral vasoconstriction syndrome, *TCCS* Transcranial color-coded duplex sonography, *y* Year(s)

^a^The percentages were calculated as subjects with menopause divided by the number of female subjects (*n* = 117 for RCVS with complete questionnaire; *n* = 35 for HCs)

### Part 1: temporal investigations

**Fig. 2:**
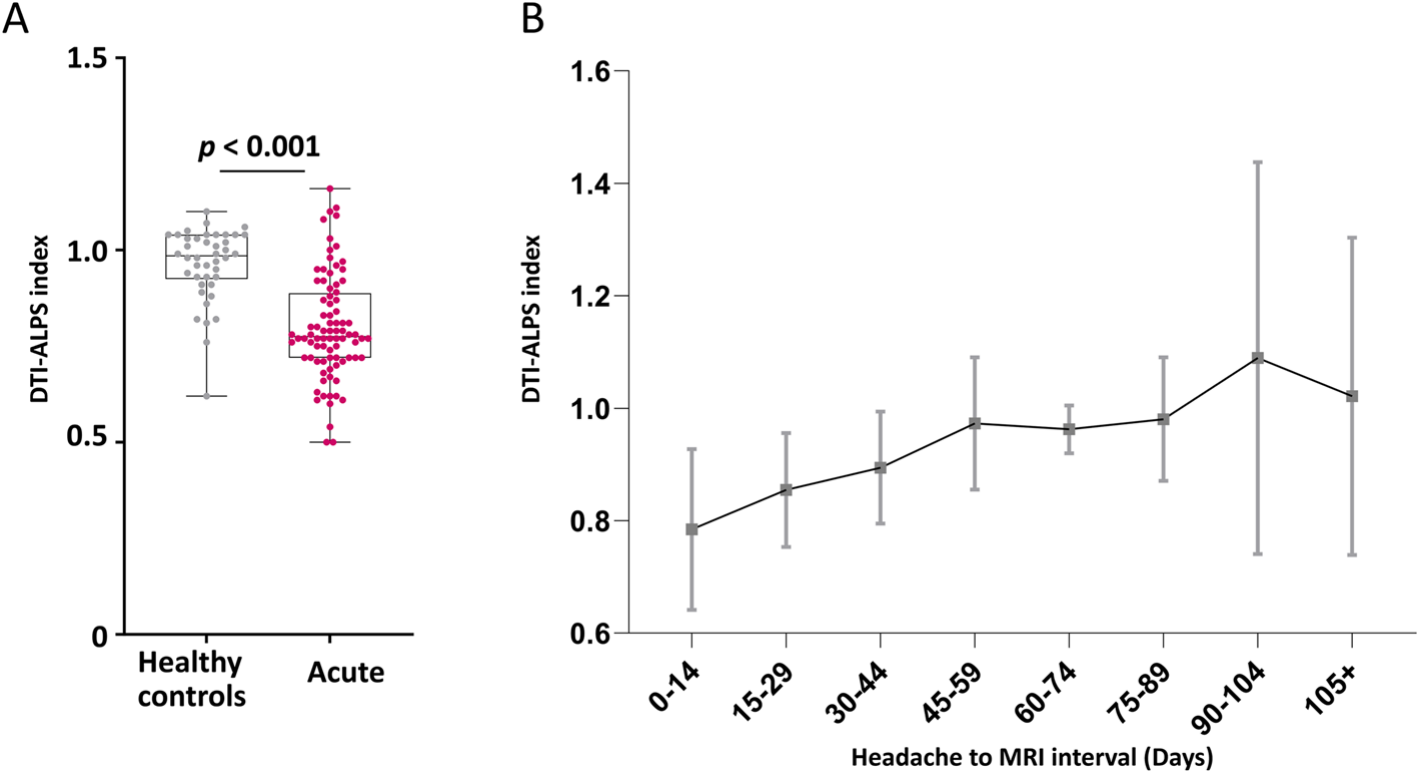
The DTI-ALPS index in RCVS subjects was lower in the acute stage and continuously increased after disease onset. **A** In the acute stage analysis, the DTI-ALPS index was lower in the acute RCVS than in healthy controls (HCs). The box and whisker plots use horizontal lines to denote the median values, boxes to denote the interquartile ranges, and whiskers to denote the minimum and maximum values. **B** The time-trend analysis, using 15 days as a time segment, demonstrated that the DTI-ALPS index continuously increased after disease onset in RCVS subjects. The gray squares indicate the mean values, and the gray lines and error bars indicate the ± 95% confidence intervals. The black line connects the mean DTI-ALPS index in each time segment

The data in the acute stage analysis in Part 1 demonstrated that the DTI-ALPS index in RCVS subjects in the acute group was significantly lower than HCs (0.80 ± 0.10; 0.78 [0] vs. 0.96 ± 0.09; 0.98 [0.11]; *p* < 0.001; Table 2 and Fig. 2A). There were no significant differences in age (*p* = 0.510) and sex (*p* = 0.725) between acute RCVS and HCs.

**Table 2:** Major results of the Parts 1 and 2

**Part 1: Temporal investigations**			
*Acute stage analysis*			
	**Healthy controls (HCs)**	**Acute group**	***p***
Number of subjects	42	84	-
Age (y)	46.0 ± 4.5	47.0 ± 12.4	0.510
Number of females	35 (83.3%)	68 (81.0%)	0.725
Disease onset to MR interval (days)	-	8.1 ± 8.6	-
DTI-ALPS index (mean ± SD; median [IQR])	0.96 ± 0.09; 0.98 [0.11]	0.80 ± 0.10; 0.78 [0]	< 0.001
*Longitudinal analysis*			
	**Acute stage**	**Remission stage**	***p***
Age (y)	49.0 ± 11.9		-
Number of females	34 (81.0%)		-
Disease onset to MR interval (days)	2.6 ± 1.2	113.9 ± 38.4	-
DTI-ALPS index (mean ± SD; median [IQR]; *n* = 42 pairs)	0.78 ± 0.15; 0.77 [0.23]	1.06 ± 0.32; 1.00 [0.38]	< 0.001
Distal ICA RI (*n* = 38 pairs)	0.56 ± 0.06; 0.56 [0.08]	0.54 ± 0.05; 0.54 [0.08]	0.048
**Part 2: Vascular investigations**^**a**^			
	Number	r_s_	*p*
DTI-ALPS index vs. distal ICA RI	131	- 0.249	0.004
DTI-ALPS index vs. mean proximal MCA flow (Disease onset to MR ≥ 50 and < 100 days)	43	0.434	0.004

*DTI-ALPS* Diffusion-tensor imaging along the perivascular space, *ICA* Internal carotid artery, *IQR* Interquartile range, *MCA* Middle cerebral artery, *MR* Magnetic resonance, *RI* Resistance index, *SD* Standard deviation

^a^Detailed results from Part 2 are listed in Supplementary Table 4 in Additional file 1

The time-trend analysis, using 15 days as a time segment, revealed a continuously increasing trend in the DTI-ALPS index after disease onset (Fig. 2B). To evaluate the dynamic change of DTI-ALPS index during the acute stage, we used smaller time segments, i.e., 3 or 4 days, to elaborate the DTI-ALPS time-trend in RCVS subjects (Supplementary Figure 2 in Additional file 1). Although there seemed to be a minor drop of DTI-ALPS index at 8–10 days after disease onset in the 3-day time segment analysis, there was only one DTI-ALPS value within this time period. This trend was not observed in the 4-day segment analysis. Hence, whether there is possible worsening of the DTI-ALPS index during the acute stage remains unclear.

The longitudinal analysis demonstrated that the DTI-ALPS index was significantly lower in the acute stage than in the remission stage (*p* < 0.001; *n* = 42) in RCVS subjects. The distal RI was significantly lower in the remission stage than in the acute stage (*p* = 0.048; *n* = 38).

### Part 2: vascular investigations

**Fig. 3:**
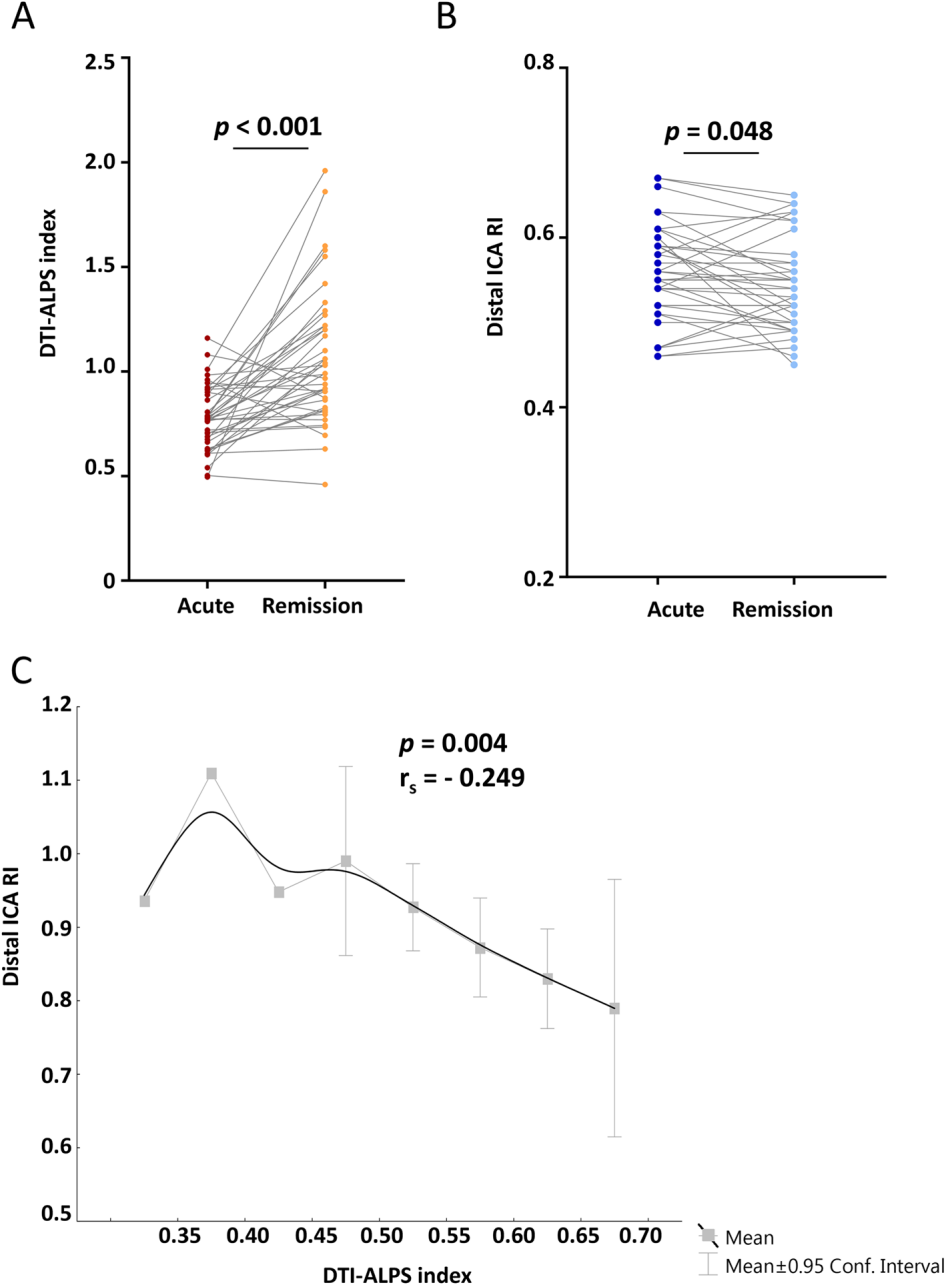
The DTI-ALPS index in RCVS subjects was higher in the remission stage than acute stage and was correlated with ICA RI. **A** The longitudinal analysis in Part 1 revealed that the DTI-ALPS index was higher in the remission stage than acute stage in RCVS subjects. **B** The values of distal ICA RI were lower in the remission than in acute RCVS. **C** A negative correlation between the DTI-ALPS index and distal ICA RI in all subjects was demonstrated (*p* = 0.004 < *q* = 0.05/11). The gray solid squares indicate the mean values, and the error bars indicate the ± 95% confidence intervals

A significantly negative correlation between distal ICA RI and DTI-ALPS index was demonstrated (*p* = 0.004 < *q* = 0.05/11, *r*_*s*_ = -0.249, Table 2 and Fig. 3C). This indicated that the DTI-ALPS index was lower when the distal ICA RI was higher. Since the slope of the curve over time deflected at approximately 50 days after disease onset (Fig. 2B), we further divided the subjects in Part 2 into groups based on onset to MR intervals of < 50 (*n* = 73), ≥ 50 and < 100 (*n* = 43), and ≥ 100 (*n* = 15) days. The DTI-ALPS index was positively correlated with the mean MCA flow velocity between 50 and 100 days, which indicated that the DTI-ALPS index was higher when the mean MCA flow velocity was also higher during this period (*p* = 0.004 < *q* = 0.05/11, *r*_*s*_ = 0.434; Supplementary Figure 3 in Additional file 1). Detailed results of the vascular investigations are shown in Supplementary Table 4 in Additional file 1.

### Part 3: clinical investigations

The total HIT-6 scores (*p* = 0.020, *r*_*s*_ = -0.215) were negatively correlated with the DTI-ALPS index (Supplementary Table 5 and Supplementary Figure 4 in Additional file 1). This indicated that the DTI-ALPS index was lower when this headache score was severe. The correlations of the DTI-ALPS index with other clinical parameters were not statistically significant (Supplementary Table 6 in Additional file 1).

Since the menopause status was negatively correlated with the DTI-ALPS index (*p* = 0.013; rs = -0.275), the DTI-ALPS differences between presence and absence of menopause were evaluated. The investigations were conducted in these selected subjects only because we could only confirm the menopause status in female subjects with complete questionnaires (*n* = 81). Among these 81 subjects, the age-adjusted differences between presence and absence of menopause regarding acute (*n* = 56; *p* = 0.163) and remission (*n* = 11; *p* = 0.856) RCVS were not significant (Supplementary Table 7 in Additional file 1).

### Potential effect of nimodipine on glymphatics

Nimodipine may potentially affect glymphatics [40, 41]. In order to delineate the potential effects of nimodipine on the glymphatic function in RCVS subjects, we further evaluated the DTI-ALPS differences between subjects with (*n* = 43) and without (*n* = 41) nimodipine during the acute stage of RCVS. The sex- and age-matched comparison depicted no significant differences of DTI-ALPS index between these 2 subgroups (*p* = 0.546; Supplementary Table 8 in Additional file 1).

### Dynamics of vasoconstriction trends

The LI was used as a vasoconstriction severity indicator in RCVS [3, 38]. Although its correlation with DTI-ALPS index was not significant (*p* = 0.446), it was important to evaluate the time-trends of vasoconstriction severity with DTI-ALPS dynamics throughout the disease course (Supplementary Figure 5 in Additional file 1). DTI-ALPS index started to increase shortly after the disease onset. In contrast, the LI reached its highest value around 2nd to 3rd week after the disease onset. Both parameters were presented with a relatively flattened trends around 2 months after the disease onset.

## Discussion

The glymphatic transport function was lower in the acute stage and then was elevated during the recovery of vasoconstriction. The DTI-ALPS index of glymphatic function was negatively correlated with the clinical HIT-6 scores. This indicated that the DTI-ALPS index was lower when this headache score (HIT-6) was severe. Remarkably, glymphatic function was associated with vasomotor controls, with DTI-ALPS results showing a negative correlation with the ICA RI in all RCVS subjects and a positive correlation with the MCA flow velocity between 50 and 100 days after disease onset. These findings indicated that the DTI-ALPS index was lower when there was higher ICA RI. However, during the subacute stage, higher DTI-ALPS was found concomitantly with higher MCA flow velocity. Together, these results underscore the importance of glymphatic dynamics in this enigmatic neurovascular disorder and provide potential mechanistic insights into the complex interaction between glymphatic transport, vasomotor control and pain modulation.

The landmark characteristic of RCVS is the reversible vasoconstriction [2]. The data in Part 1 revealed decreased DTI-ALPS index in acute stage than in HCs and remission stage. These findings suggest glymphatic dysfunction in the acute stage and the potential reversibility of the glymphatic functions in the remission stage. Previous studies depicted higher levels of 8-iso-prostaglandin F_2α_, an oxidative stress marker with potent vasoconstrictive property, in patients with RCVS during the acute stage [6]. Metabolomics analysis also indicated the potential pathogenic role of oxidative stress in RCVS [42]. In addition, prominent blood–brain barrier disruption as indicated by MR-detectable gadolinium-based contrast agent leakage into the paravascular glymphatic spaces have been noted in RCVS [7–9]. Therefore, with the data in Part 1, we speculated the relatively decreased glymphatic flow in acute stage might exacerbate the accumulation of the vasoconstrictors and reactive oxygen species intracranially, which were brought by the blood–brain barrier disruption in the acute stage. This phenomenon would further cause severer vasoconstrictions. Furthermore, the data in the time-trend investigations with a continuously increasing DTI-ALPS index trend in RCVS after disease onset was parallel to the reversible nature of the vasoconstriction in RCVS that the gradually improved glymphatic flow may remove the vasoconstrictors and reactive oxygen species out of the cranium, subsequently contributes to the improvement of the vasoconstrictions along the disease course. The finding that the DTI-ALPS index started to increase prior to the improvement of LI may suggest that during the acute stage, partial improvement of the glymphatic function was insufficient to counteract the effects of perivascular vasoconstrictors. The flattening trends of both DTI-ALPS and LI 2 months after disease onset may indicate the gradual recovery of the potential physiological coupling of glymphatic function and vasomotion. Interestingly, our data may also align with the findings of a previous study, which demonstrated a dynamic change in WMHs in RCVS [34] since the WMH formation was strongly associated with glymphatic dysfunction and impaired cerebrospinal fluid clearance in other neurological disorders [43].

The vascular investigations revealed negative correlations between RI of the distal ICA on TCCS and the DTI-ALPS index in all RCVS subjects. The RI reflected the vascular distal resistance and vascular stiffness [44]. Since stenosis of the extracranial ICA is not common in RCVS, we believe that the increased RI values may partially indicate increased distal resistance due to intracranial vasoconstriction [45]. We speculated that the vasoconstriction and autonomic dysfunction [5] in RCVS may have contributed to the decreased pulsatility [46] of distal vessels, which may further dampen glymphatic flow [47]. In fact, arterial pulsation is considered one of the potential driving forces of glymphatic flow and is reduced in subjects with cardiovascular diseases, including hypertension [47]. Additionally, dysregulated autonomic functions, i.e., sympathetic overactivity and parasympathetic hypofunction, in the acute stage of RCVS [5] may contribute to impaired glymphatic function, as increased sympathetic activity has been found to impair glymphatic transport [28]. Moreover, the positive correlation between mean MCA flow and the DTI-ALPS index between 50 and 100 days after disease onset supported that the physiological coupling of glymphatics and vasomotor control may be disrupted [47] during the acute stage and restored in the later stage of the disease. After the improvement of vasoconstriction and autonomic dysfunction, the physiologic correlation between vascular flow and glymphatics then emerged.

The negative correlations between HIT-6 scores demonstrated that there may be clinical correlates of glymphatic functions in subjects with RCVS. The HIT-6 measured the daily life impacts (Supplementary Table 2 in Additional file 1) due to RCVS. A recent study revealed that the DTI-ALPS index may be an imaging marker of cancer pain [48]. Our data further support the potential use of the DTI-ALPS index in the evaluation of subjects with RCVS regarding clinical impacts and implicate the potential role of glymphatics in pain modulation. Several recent studies also enhanced the roles of glymphatic system in headache disorders (Supplementary Table 9 in Additional file 1) [19, 31, 32, 49–55]. Nevertheless, since some studies depicted normal glymphatic functions in certain headache disorders, including episodic migraine [19, 52] and new daily persistent headache, [24] and some demonstrated contradictory findings, [32] the potential mechanism of glymphatics in pain or headache modulation remains to be explored.

Although the diagnosis of RCVS majorly relies on the vascular reversibility and clinical presentations, our study indicates that the “DTI-ALPS index reversibility” may be a unique disease feature of RCVS and may serve as an ancillary imaging marker. However, its potential role as a diagnostic marker remains to be explored.

### Strengths and limitations

This study had several strengths. This study successfully demonstrated dynamic changes in glymphatic functions in subjects with complex neurovascular disorders. The significantly lower DTI-ALPS indices in the acute stage and gradually increased values as the disease resolved provided evidence that glymphatic function fluctuates with RCVS disease course. This large-scale study was based on DTI data, which were acquired noninvasively and did not require gadolinium-based contrast agent administration. All imaging was performed on the same MRI machine with sophisticated imaging and preprocessing protocols. The data processing was performed after software-based motion corrections and synchronization. Furthermore, we marked the ROIs meticulously and avoided the registrations of ROIs onto regions with potential influences in the DTI data, including areas with WMHs or overt vascular structures. The good interrater reliability supports the credibility of our methods. However, some limitations existed. In our practice, all RCVS subjects were treated with nimodipine upon diagnosis since it was not ethical to leave the patients untreated [37]. Therefore, despite the fact that the DTI-ALPS index values in acute RCVS between subjects with and without nimodipine were similar and we monitored all the subjects closely to avoid any hypotensive episodes with systolic blood pressure < 100 mmHg, [38] the effects of nimodipine on the hemodynamics and glymphatics may still exist. In fact, some studies have indicated that the nimodipine could increase glymphatic flow [40, 41]. One recent preclinical animal model demonstrated that nimodipine could increase the glymphatic functions and decrease neurological deficits in mice with experimental subarachnoid hemorrhage [41]. Therefore, it is possible that nimodipine has contributed to the incremental trend of DTI-ALPS index in our patients along the disease course. Further studies are needed to investigate whether nimodipine could accelerate the recovery of glymphatic dysfunction in RCVS. In this study, we did not exhaustively evaluate potentially glymphatics-related comorbidities in our patients, including posttraumatic headache, [51, 56] obstructive sleep apnea [57] or increased intracranial pressures [56, 58]. Therefore, the potential glymphatic influence by these disorders could not be completely excluded.

## Conclusions

Glymphatic function was altered dynamically in subjects with RCVS in a distinct manner that has never been observed in other diseases. The impaired glymphatic functions may be correlated with dysfunctional vasomotor control, while the physiological coupling between vasomotor control and glymphatic functions may be partially restored during disease recovery.

### Supplementary Information

**Additional file 1.**

## Data Availability

The datasets used and/or analysed during the current study are available from the corresponding author on reasonable request.

## Abbreviations

DTI: Diffusion tensor imaging
DTI-ALPS: Diffusion tensor imaging along the perivascular space
HC: Healthy control
HIT-6: The Six-item headache Impact Test
ICA: Internal carotid artery
IQR: Interquartile range
LI: Lindegaard index
MCA: Middle cerebral artery
MRI: Magnetic resonance imaging
PI: Pulsatility index
RCVS: Reversible cerebral vasoconstriction syndrome
RI: Resistance index
ROI: Regions of interest
SD: Standard deviation
TCCS: Transcranial color-coded duplex sonography
WMH: White matter hyperintensity
