# Supplementary material for: Dynamic changes in glymphatic function in reversible cerebral vasoconstriction syndrome
Source: J Headache Pain. 2024 Feb 5;25(1):17. doi: 10.1186/s10194-024-01726-1 (PMC10840154; doi:10.1186/s10194-024-01726-1)
Supplement: Supplementary file 1 — Additional file 1. [file 10194_2024_1726_MOESM1_ESM.docx]

**Additional file 1: Supplementary Materials**

**Supplementary Table 1. Inclusion and exclusion criteria of this study**

| **For reversible cerebral vasoconstriction syndrome (RCVS) subjects** | |
| --- | --- |
| **Inclusion criteria** | 1. Subjects fulfilling the RCVS diagnosis criteria^a^ A. With 2 or more severe thunderclap headache with or without focal neurological deficits  B. Vasoconstriction depicted on magnetic resonance angiography (MRA)  C. Vasoconstriction reversibility by follow-up MRA  D. Aneurysmal subarachnoid hemorrhage or other intracranial disorders should be excluded by appropriate investigations^b^ |
| **Exclusion criteria** | 1. Currently under any medications other than nimodipine  2. The follow-up MRA shows no reversibility of vasoconstriction^c^ |
| **For healthy controls** | |
| **Exclusion criteria** | 1. Known history of malignancy (not limited to intracranial cancer)  2. Psychiatric disorders  3. Major neurological diseases, including but not limited to ischemic stroke, hemorrhagic stroke, demyelination, or neuroinflammatory diseases, intracranial tumors, and neurodegenerative diseases  4. Personal history of moderate to severe migraine-like headache  5. Family history of moderate to severe migraine-like headache. The given family member is within third degree relatives. |
| **For all study subjects** | |
| **Exclusion criteria** | 1. Suboptimal imaging quality, including but not limited to metallic artifacts, incomplete imaging, or any potential factors affecting imaging analysis  2. Intracranial tumors  3. Any factors that are not suitable or safe for MRI exams, including but not limited to claustrophobia, large tattoo, insulin pump, glucose sensor, with any metallic implants/foreign body  4. Previous craniotomy/craniectomy  5. Pregnancy  6. Subjects do not wish to continue |

^a^The RCVS diagnostic criteria in this study was based on the criteria used in the previous studies.(1-3) These criteria were consistent with those in the International Classification of Headache Disorders, third version (ICHD-3).

^b^Related changes of RCVS, including posterior reversible encephalopathy syndrome, ischemic stroke, intracerebral hemorrhage or cortical subarachnoid hemorrhage are allowed for the RCVS diagnosis.

^c^Since RCVS subjects may be enrolled at the acute stage, the follow-up MRA may be conducted after enrollments. If the follow-up MRA does not depict the reversibility of vasoconstriction, the subject shall be excluded.

1. Chen SP, Chou KH, Fuh JL, Huang YH, Huang CC, Lirng JF, Wang YF, Lin CP, Wang SJ. Dynamic Changes in White Matter Hyperintensities in Reversible Cerebral Vasoconstriction Syndrome. JAMA Neurol 2018;75(9):1106-1113. doi: 10.1001/jamaneurol.2018.1321

2. Wu CH, Lirng JF, Ling YH, Wang YF, Wu HM, Fuh JL, Lin PC, Wang SJ, Chen SP. Noninvasive Characterization of Human Glymphatics and Meningeal Lymphatics in an in vivo Model of Blood-Brain Barrier Leakage. Ann Neurol 2021;89(1):111-124. doi: 10.1002/ana.25928

3. Wu CH, Lirng JF, Wu HM, Ling YH, Wang YF, Fuh JL, Lin CJ, Ling K, Wang SJ, Chen SP. Blood-Brain Barrier Permeability in Patients With Reversible Cerebral Vasoconstriction Syndrome Assessed With Dynamic Contrast-Enhanced MRI. Neurology 2021;97(18):e1847-e1859. doi: 10.1212/WNL.0000000000012776

4. International Headache Society (IHS). Headache Classification Committee of the International Headache Society (IHS) The International Classification of Headache Disorders, 3rd edition. Cephalalgia 2018;38(1):1-211. doi: 10.1177/0333102417738202

**Supplementary Table 2. The Six-Item Headache Impact Test (HIT-6)**

| **Questions** | **Never** | **Rarely** | **Sometimes** | **Very often** | **Always** |
| --- | --- | --- | --- | --- | --- |
| **(A) When you have headaches, how often is the pain severe?** | 6 | 8 | `10 | 11 | 13 |
| **(B) How often do headaches limit your ability to do usual daily activities?** | 6 | 8 | 10 | 11 | 13 |
| **(C) When you have a headache, how often do you wish you could lie down?** | 6 | 8 | 10 | 11 | 13 |
| **(D) In the past 4 weeks, how often have you felt too tired to do work or daily activities because of your headaches?** | 6 | 8 | 10 | 11 | 13 |
| **(E) In the past 4 weeks, how often have you felt fed up or irritated because of your headaches?** | 6 | 8 | 10 | 11 | 13 |
| **(F) In the past 4 weeks, how often did headaches limit your ability to concentrate on work or daily activities?** | 6 | 8 | 10 | 11 | 13 |
| **Total scores = (A) + (B) + (C) + (D) + (E) + (F)** | | | | | |

**Supplementary Table 3. Definition of the calculated vascular parameters on the transcranial color-coded duplex sonography (TCCS)**

| **Mean middle cerebral artery (MCA) pulsatility index (MMPI)^a^** | Mean PI values in proximal and distal segments^b^ of both MCAs |
| --- | --- |
| **Mean MCA resistance index (MMRI)^a^** | Mean RI values in proximal and distal segments of both MCAs |
| **Mean MCA resistance index ratio (MRIR)** | Ratio of distal to proximal RI |
| **Lindegaard index (LI)** | Ratio of the mean flow velocity of the MCA to that of the ipsilateral ICA. |

^a^Since the PIs in large vessels decrease from proximal to distal segments(1) and are affected by the locations of vasoconstrictions, the MMPI was calculated as the mean values of both segments. The MMRI was calculated in the similar methods.(2, 3)

^b^The MCA evaluations included proximal and distal M1 segments separated by a 20-mm distance.(2, 3)

1. Zarrinkoob L, Ambarki K, Wahlin A, Birgander R, Carlberg B, Eklund A, Malm J. Aging alters the dampening of pulsatile blood flow in cerebral arteries. J Cereb Blood Flow Metab 2016;36(9):1519-1527. doi: 10.1177/0271678X16629486

2. Chen SP, Fuh JL, Chang FC, Lirng JF, Shia BC, Wang SJ. Transcranial color doppler study for reversible cerebral vasoconstriction syndromes. Ann Neurol 2008;63(6):751-757. doi: 10.1002/ana.21384

3. Wu CH, Lirng JF, Wu HM, Ling YH, Wang YF, Fuh JL, Lin CJ, Ling K, Wang SJ, Chen SP. Blood-Brain Barrier Permeability in Patients With Reversible Cerebral Vasoconstriction Syndrome Assessed With Dynamic Contrast-Enhanced MRI. Neurology 2021;97(18):e1847-e1859. doi: 10.1212/WNL.0000000000012776

**Supplementary Table 4. Detailed Results of Part 2**

| **Vascular parameters on TCCS** | **r_s_** | ***p*** |
| --- | --- | --- |
| *All participants (n = 131).* | | |
| Distal M1 PI | 0.066 | 0.454 |
| Proximal M1 PI | 0.053 | 0.548 |
| Distal M1 RI | - 0.032 | 0.749 |
| Proximal M1 RI | - 0.135 | 0.150 |
| Distal ICA RI | - 0.249 | 0.004 |
| Mean proximal MCA flow | - 0.058 | 0.538 |
| Mean distal ICA flow | - 0.110 | 0.211 |
| MMPI | - 0.133 | 0.184 |
| MMRI | - 0.070 | 0.489 |
| MRIR | - 0.045 | 0.658 |
| LI | 0.072 | 0.446 |
| *Disease onset to MR < 50 days (n = 73)* | | |
| Distal M1 PI | - 0.172 | 0.209 |
| Proximal M1 PI | - 0.199 | 0.112 |
| Distal M1 RI | - 0.130 | 0.343 |
| Proximal M1 RI | - 0.200 | 0.109 |
| Distal ICA RI | - 0.260 | 0.026 |
| Mean proximal MCA flow | 0.210 | 0.093 |
| Mean distal ICA flow | 0.035 | 0.767 |
| MMPI | - 0.178 | 0.194 |
| MMRI | - 0.158 | 0.248 |
| MRIR | - 0.108 | 0.431 |
| LI | 0.138 | 0.272 |
| *Disease onset to MR ≥ 50 and < 100 days (n = 43)* | | |
| Distal M1 PI | 0.076 | 0.653 |
| Proximal M1 PI | 0.050 | 0.772 |
| Distal M1 RI | 0.074 | 0.664 |
| Proximal M1 RI | 0.083 | 0.624 |
| Distal ICA RI | - 0.228 | 0.141 |
| Mean proximal MCA flow | 0.434 | 0.004 |
| Mean distal ICA flow | 0.004 | 0.978 |
| MMPI | 0.027 | 0.878 |
| MMRI | 0.014 | 0.934 |
| MRIR | 0.006 | 0.973 |
| LI | 0.136 | 0.423 |
| *Disease onset to MR ≥ 100 days (n = 15)* | | |
| Distal M1 PI | 0.273 | 0.446 |
| Proximal M1 PI | 0.044 | 0.886 |
| Distal M1 RI | 0.273 | 0.446 |
| Proximal M1 RI | 0.055 | 0.859 |
| Distal ICA RI | - 0.411 | 0.128 |
| Mean proximal MCA flow | - 0.195 | 0.523 |
| Mean distal ICA flow | 0.171 | 0.541 |
| MMPI | 0.333 | 0.347 |
| MMRI | 0.333 | 0.347 |
| MRIR | - 0.200 | 0.580 |
| LI | - 0.096 | 0.754 |

LI = Lindegaard index; ICA = Internal carotid artery; MCA = Middle cerebral artery; MMPI = Mean middle cerebral artery pulsatility index; MMRI = Mean middle cerebral artery resistance index; MRIR = Middle cerebral artery resistance index ratio; MRI = Magnetic resonance; PI = Pulsatility index; RI = Resistance index; TCCS = Transcranial color-coded duplex sonography

**Supplementary Table 5. Correlation results the Clinical investigations regarding HIT-6 scores and DTI-ALPS index**

|  | HIT-6 scores | r_s_ (95% confidence intervals) | *p* |
| --- | --- | --- | --- |
| DTI-ALPS index  vs. HIT-6 scores (n = 117) | 57.4 ± 9.0 | - 0.215 (- 0.386 – - 0.029) | 0.020 |

DTI-ALPS = Diffusion-tensor imaging along the perivascular space; HIT-6 = Six-item Headache Impact Test

**Supplementary Table 6. Detailed Results of Part 3**

| **Variables^a^** | **Correlation coefficients (r_s_)** | ***p*** |
| --- | --- | --- |
| Gender^b^ | 0.053 | 0.572 |
| Age^b^ | -0.053 | 0.571 |
| Menopause^c^ (n = 45) | -0.275 | 0.013 |
| **Headache triggers** | | |
| Exertion (n = 17) | -0.145 | 0.116 |
| Defecation (n = 47) | 0.018 | 0.846 |
| Showering (n = 45) | -0.156 | 0.091 |
| Rage (n = 16) | -0.111 | 0.228 |
| Singing (n = 9) | -0.131 | 0.156 |
| Coughing (n = 12) | -0.155 | 0.092 |
| Sexual activity (n = 8) | -0.113 | 0.219 |
| **Accompanying symptoms** | | |
| Nausea/vomiting (n = 26) | -0.109 | 0.238 |
| Neck stiffness (n = 29) | -0.105 | 0.254 |

^a^The total number of subjects with complete questionnaires was 117.

^b^For gender and age, all subjects in this study (n = 138) were evaluated.

^c^Only evaluated in female subjects (n = 81).

**Supplementary Table 7. DTI-ALPS index analysis between presence and absence of menopause in acute and remission groups^a^**

|  | **Menopause (+)** | **Menopause (-)** | ***p*** |
| --- | --- | --- | --- |
| Acute group (n = 56) | | | |
| Number of subjects | 31 | 25 | - |
| Age (y) | 52.1 ± 11.3 | 37.0 ± 7.5 | < 0.001 |
| DTI-ALPS index (mean ± SD; median [IQR]) | 0.73 ± 0.12; 0.74 [0.16] | 0.80 ± 0.09; 0.79 [0.14] | 0.163 |
| Remission group (n = 11) | | | |
| Number of subjects | 9 | 2 | - |
| Age (y) | 53.1 ± 7.9 | 40.0 ± 14.1 | 0.090 |
| DTI-ALPS index (mean ± SD; median [IQR]) | 0.80 ± 0.15; 0.76 [0.21] | 0.87 ± 0.08; 0.87 [-] | 0.856 |

^a^Only female subjects with complete questionnaires were eligible for the analysis (n = 81).

**Supplementary Table 8. Nimodipine analysis in acute RCVS subjects**

|  | **With nimodipine** | **Without nimodipine** | ***p*** |
| --- | --- | --- | --- |
| Number of subjects | 43 | 41 | - |
| Age (y) | 49.0 ± 13.6 | 45.4 ± 10.6 | 0.178 |
| Number of females (%) | 35 (81.4%) | 37 (90.2%) | 0.879 |
| DTI-ALPS index (mean ± SD; median [IQR]) | 0.81 ± 0.13; 0.77 [0.16] | 0.80 ± 0.14; 0.79 [0.20] | 0.546 |

**Supplementary Table 9. Recent publications focusing on glymphatic functions in headache disorders**

|  | Headache disorders | Study models | Summary |
| --- | --- | --- | --- |
| Huang, W, et al. (2023) [31] | Chronic migraine | Mice | Glymphatic dysfunctions may be present in chronic migraine. |
| Lee, DA, et al. (2022) [52] | Episodic migraine | Humans | Glymphatic dysfunctions may not be present in episodic migraine. |
| Ornello R, et al. (2023) [55] | Migraine | Humans | The glymphatic functions may not be different between migraine with and without white matter hyperintensities. |
| Wu CH, et al. (2023) [19] | Episodic and chronic migraine | Humans | Glymphatic dysfunctions may be present in chronic migraine, but not be apparent in episodic migraine. |
| Yuan Z, et al. (2023) [50] | Migraine | Humans | Glymphatic dysfunctions may be present in migraine. |
| Zhang X, et al. (2023) [32] | Episodic and chronic migraine | Humans | Higher glymphatic functions may be present in chronic migraine than in episodic migraine. |
| Zhang X, et al. (2023) [49] | New daily persistent headache | Humans | Glymphatic dysfunctions may not be apparent in new daily persistent headache. |
| Kim J, et al. (2022) [53] | Cluster headache | Humans | Glymphatic dysfunctions may be present in cluster headache. |
| Yi T, et al. (2022) [54] | Headache and sleep | (Reviews) | Glymphatic system may bridge the sleep disorders with headache disorders. |
| Piantino J, et al. (2019) [51] | Posttraumatic headache | (Reviews) | Traumatic brain injury may link the posttraumatic headache to glymphatic dysfunctions. |


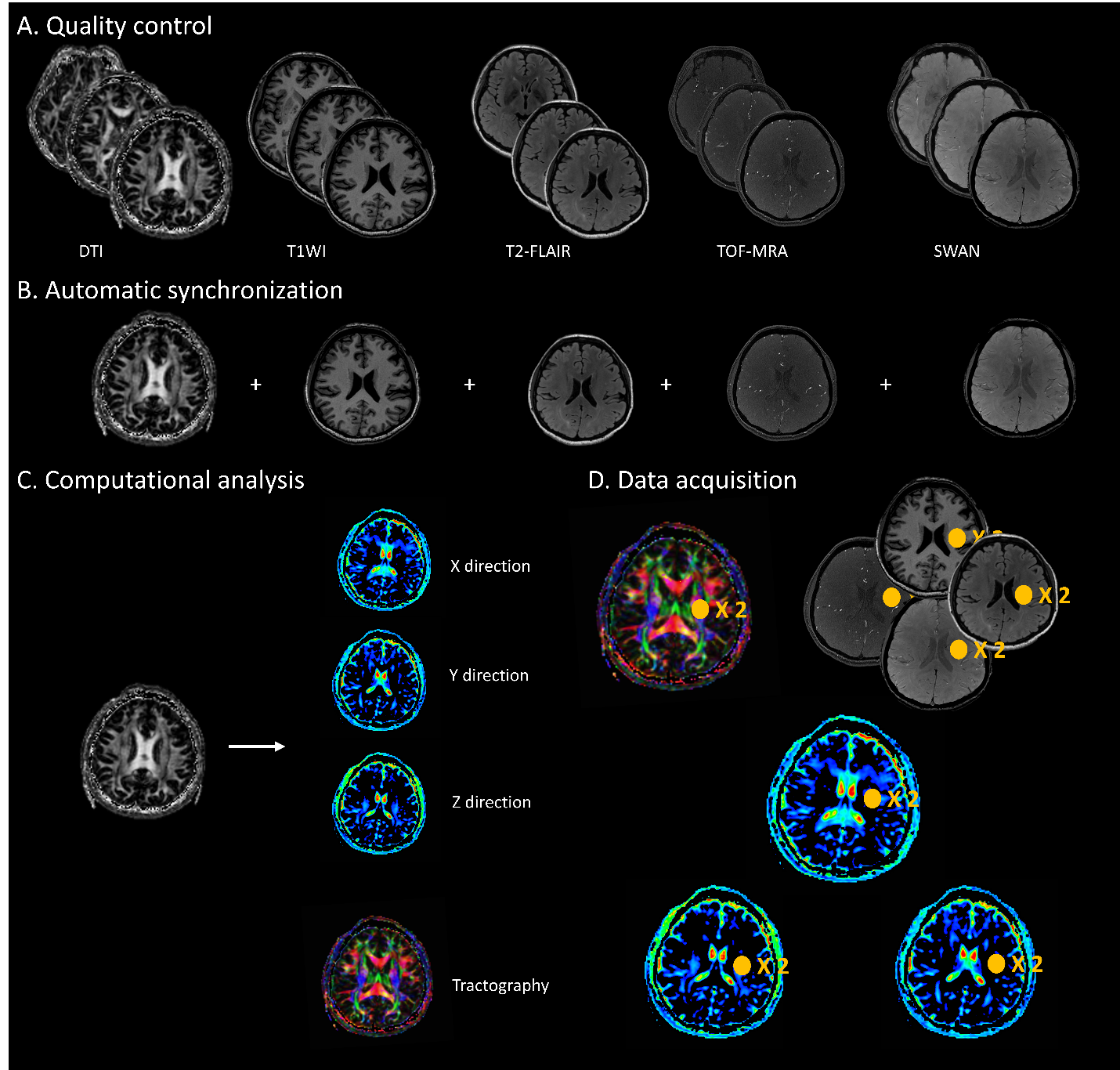


**Supplementary Figure 1. MRI data processing pipeline in this study**

(A) All the MR data used in this study were carefully inspected on both SmariIris and VolumeViewer platforms. Data with suboptimal imaging quality would be excluded. (B) After initial quality control, the data were then sent to the VolumeViewer platform and were automatically synchronized. Software-based motion correction was also conducted. (C) We then used the DTI data to extract the diffusivity maps in the x, y and z directions and the tractography map were generated. (D) Based on the tractography map, we labeled the regions of interest (ROIs) meticulously onto the projection and association areas (illustrated as “X2” as two ROIs) and acquired the mean diffusivity values within the ROIs in the x, y and z directions by the automatic registration. All the other vascular and structural MR images were used as references to avoid inclusions of apparent vascular structures or T2 hyperintensities into the ROIs. After acquisition of the x-, y- and z- diffusivities in the projection and association areas, we then calculated the values of DTI-ALPS index.


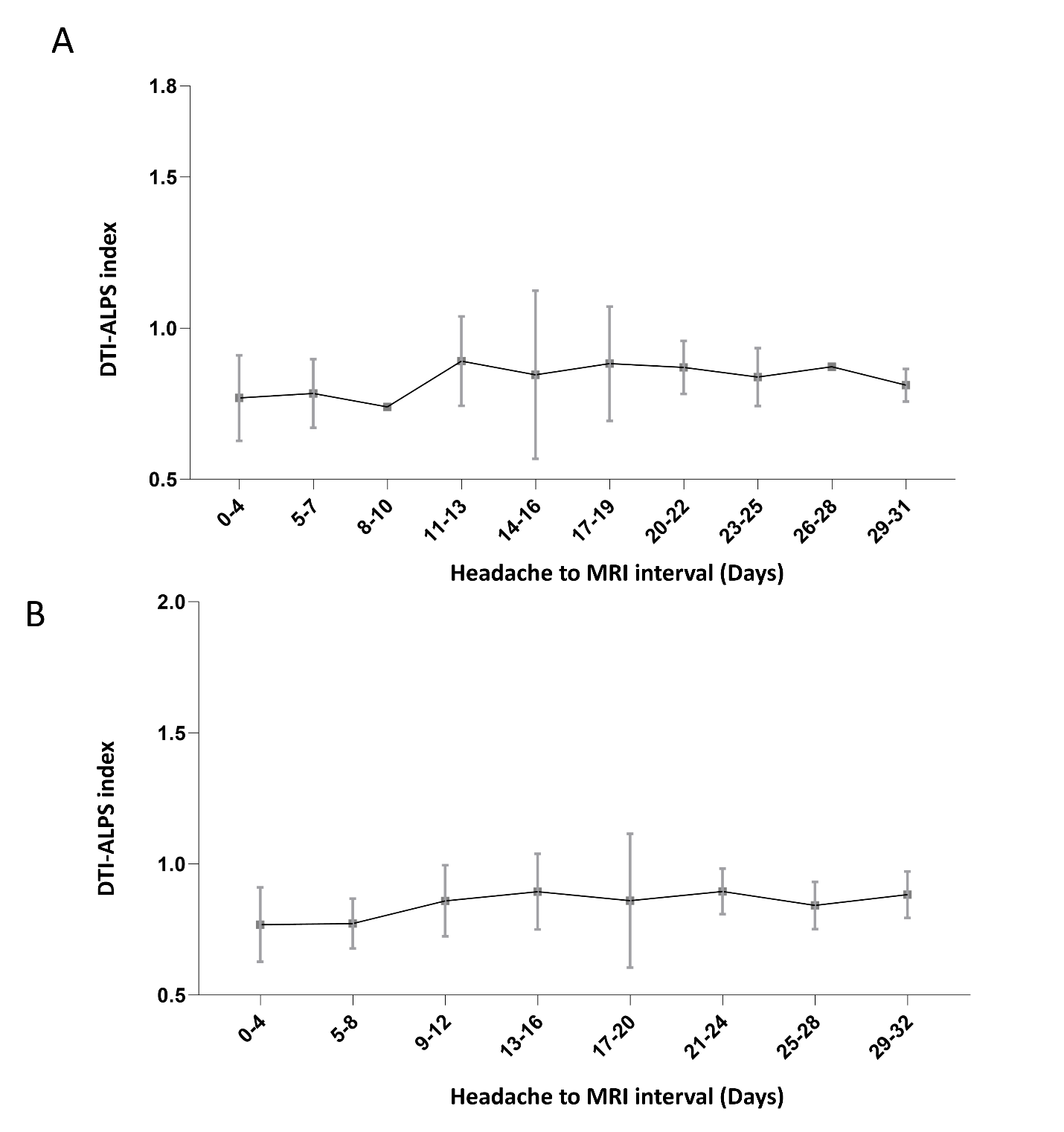


**Supplementary Figure 2. Time-trend analysis with 3 and 4 days as time segments during the acute RCVS**

Time-trend analysis was also performed using (A) 3 and (B) 4 days as time segments. The interrupted data was due to the lack of subjects within the time segment. The gray squares indicate the mean values, and the gray lines and error bars indicate the ±95% confidence intervals. The black lines connect the mean values of DTI-ALPS index in each time segment.


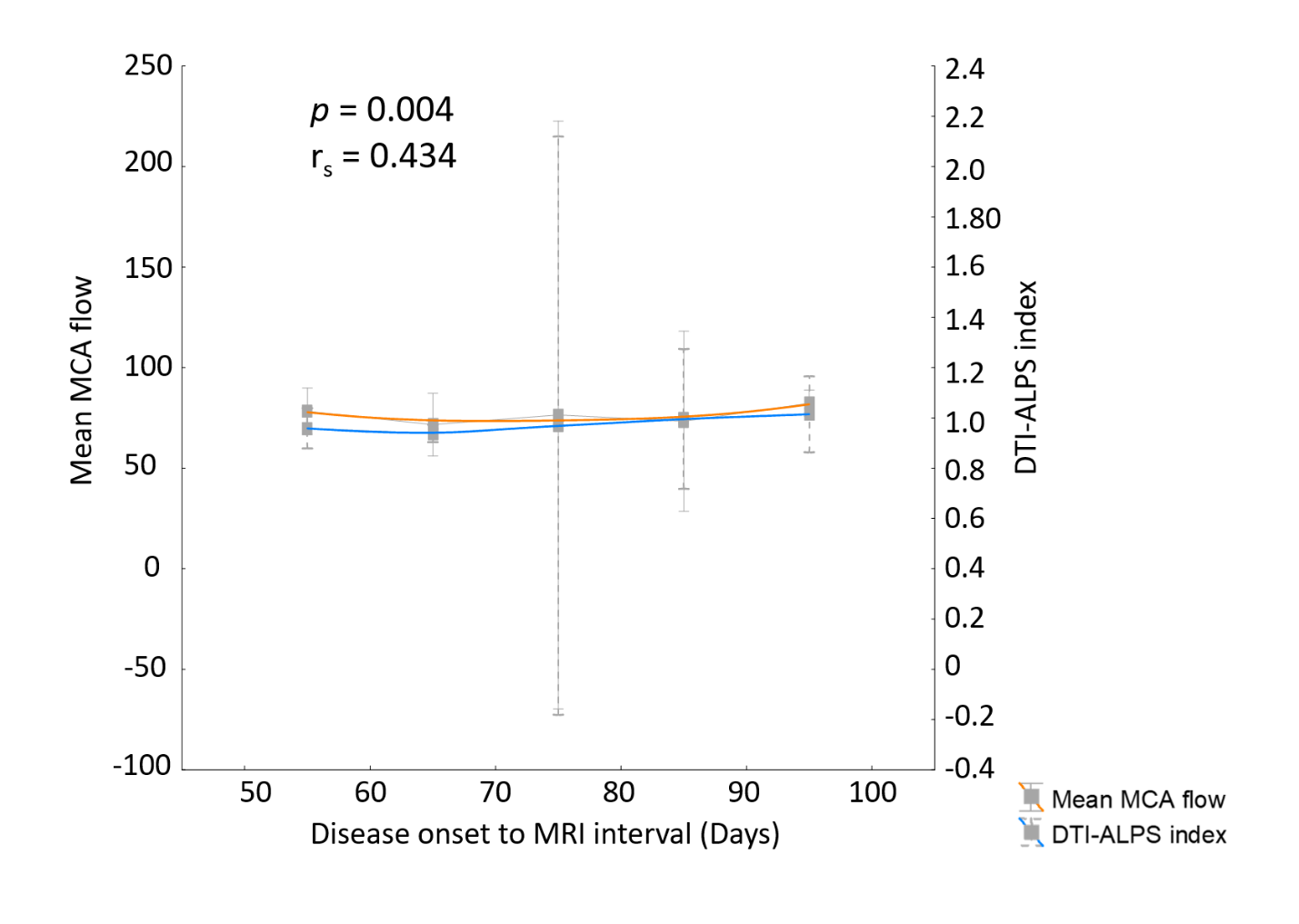


**Supplementary Figure 3. The correlation between DTI-ALPS index and mean MCA flow in subjects with disease onset to MRI intervals between 50 and 100 days.**

A positive correlation between the DTI-ALPS index and mean MCA flow in subjects with disease onset to MRI intervals from 50 to 100 days is depicted. The gray solid squares indicate the mean values, and the error bars indicate the 95% confidence intervals. The orange lines denote the mean MCA flow, and the blue line denotes the DTI-ALPS index.


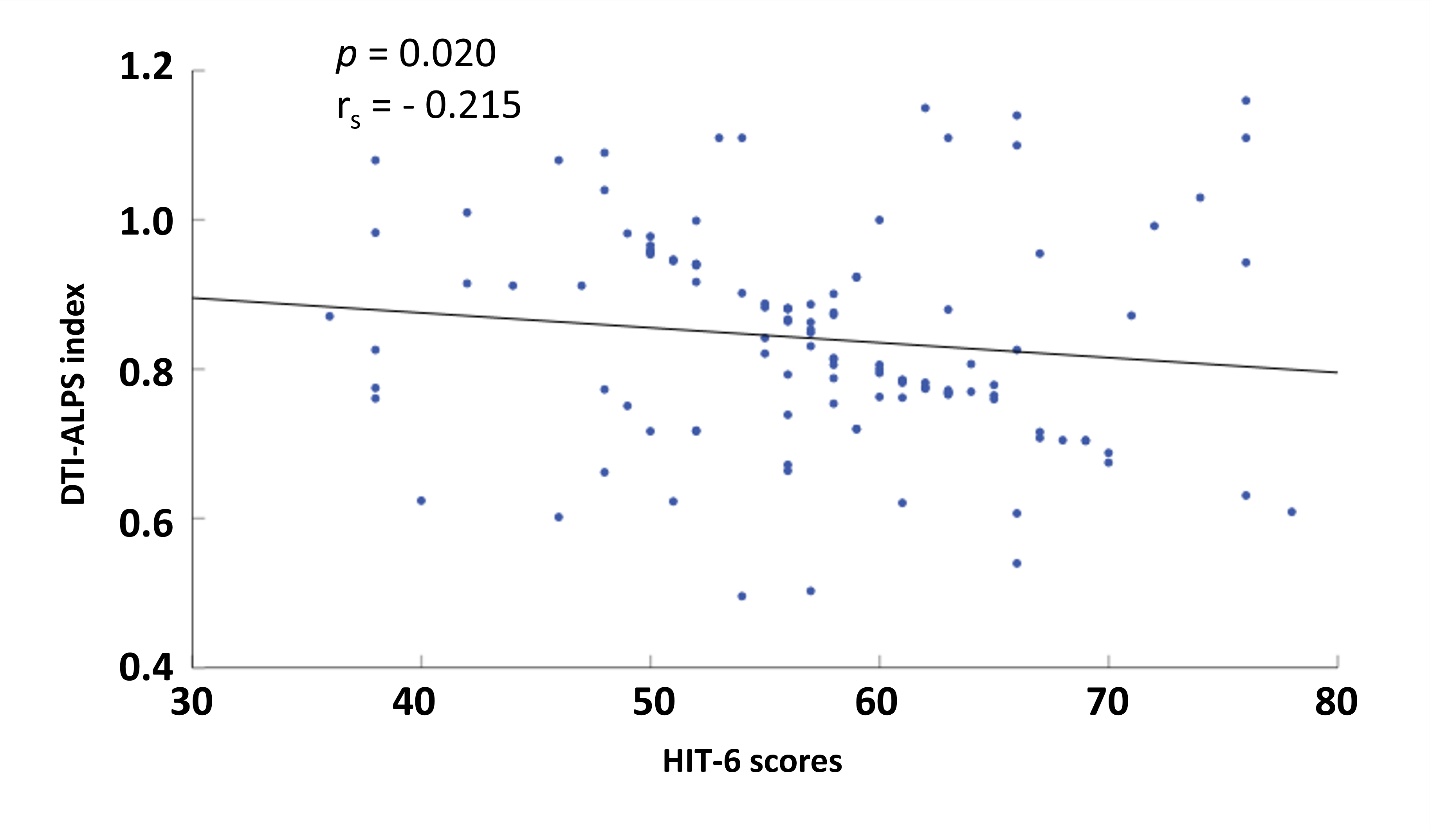


**Supplementary Figure 4. Major results of the clinical investigations.**

Part 3 revealed a negative correlation between the DTI-ALPS index and HIT-6 scores. The blue dots indicate all the values of DTI-ALPS index and HIT-6 scores. The black line indicates the fitting line of the data. The detailed questionnaire and scoring methods of the six-item Headache Impact Test (HIT-6) are shown in Supplementary Table 2 and detailed data were listed in Supplementary Table 5


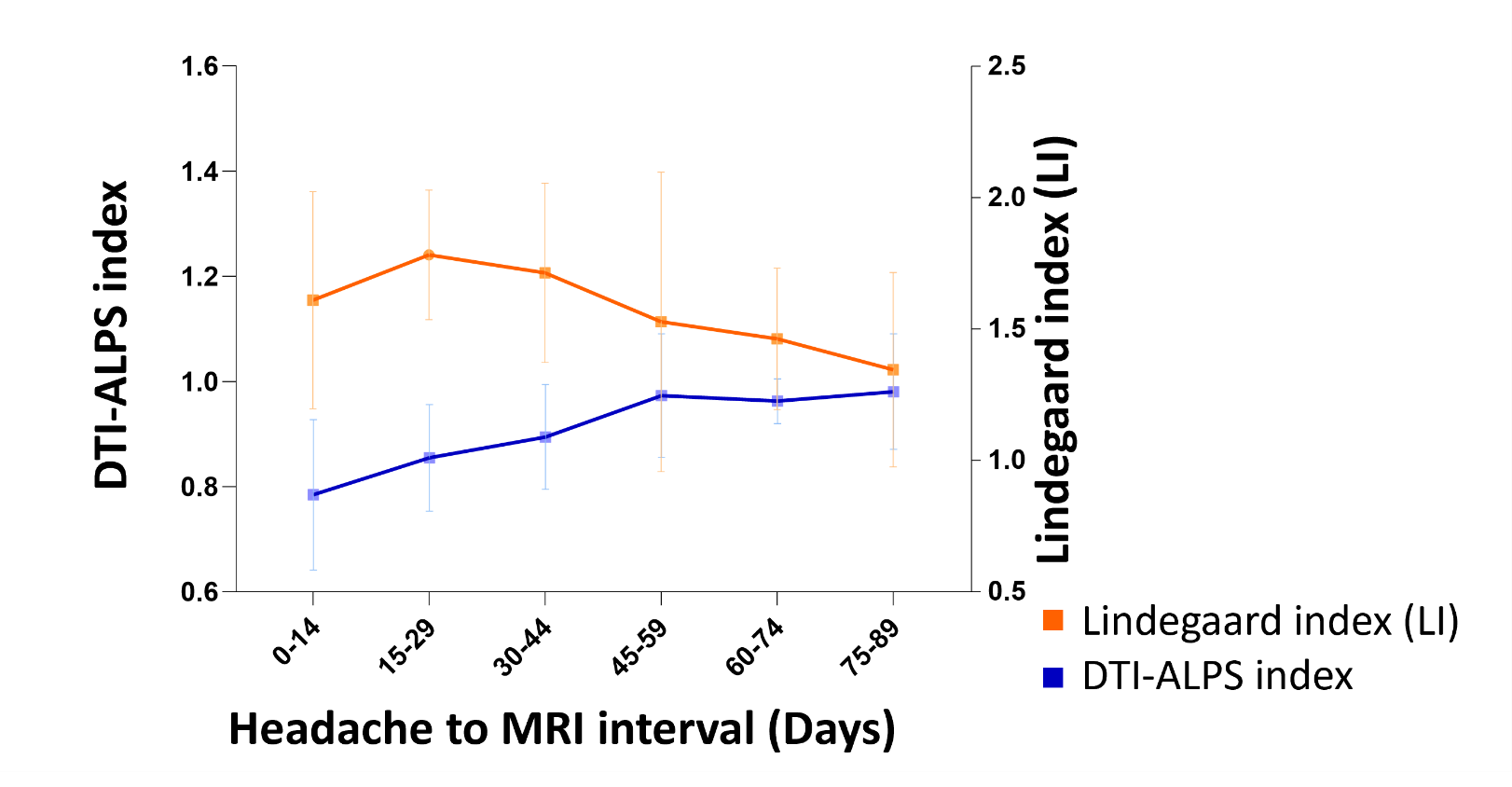


**Supplementary Figure 5. Time-trend curves of Lindegaard index and DTI-ALPS index.**

The time-trends of both parameters indicated that the DTI-ALPS index started to increase when LI vasoconstriction was relatively prominent but not the severest during the disease course. Both parameters demonstrated flattened trends around 2 months after the disease onset. The squares indicate the mean values, and the light-colored lines and error bars indicate the ±95% confidence intervals. The solid line connects the mean DTI-ALPS index or LI in each time segment.
